# Gametocyte Dynamics and the Role of Drugs in Reducing the Transmission Potential of *Plasmodium vivax*

**DOI:** 10.1093/infdis/jit261

**Published:** 2013-06-12

**Authors:** Nicholas M. Douglas, Julie A. Simpson, Aung Pyae Phyo, Hadjar Siswantoro, Armedy R. Hasugian, Enny Kenangalem, Jeanne Rini Poespoprodjo, Pratap Singhasivanon, Nicholas M. Anstey, Nicholas J. White, Emiliana Tjitra, Francois Nosten, Ric N. Price

**Affiliations:** 1 Global Health Division, Menzies School of Health Research, Charles Darwin University, Darwin 0811, Australia; 2 Centre for Tropical Medicine, Nuffield Department of Clinical Medicine, University of Oxford, United Kingdom; 3 Centre for Molecular, Environmental, Genetic and Analytic Epidemiology, University of Melbourne, Victoria, Australia; 4 Shoklo Malaria Research Unit, Mae Sod, Tak, Thailand; 5 National Institute of Health Research and Development, Jakarta; 6 Mimika District Health Authority, Timika, Papua; 7 The Menzies-National Institute of Health Research and Development Malaria Research Programme, Timika, Papua, Indonesia; 8 Faculty of Tropical Medicine, Mahidol University, Bangkok, Thailand; 9 Division of Medicine, Royal Darwin Hospital, Darwin, Australia

**Keywords:** *Plasmodium vivax*, gametocytes, antimalarial drugs, epidemiology, transmission

## Abstract

***Background.*** Designing interventions that will reduce transmission of vivax malaria requires knowledge of *Plasmodium vivax* gametocyte dynamics.

***Methods.*** We analyzed data from a randomized controlled trial in northwestern Thailand and 2 trials in Papua, Indonesia, to identify and compare risk factors for vivax gametocytemia at enrollment and following treatment.

***Results.*** A total of 492 patients with *P. vivax* infections from Thailand and 476 patients (162 with concurrent falciparum parasitemia) from Indonesia were evaluable. Also, 84.3% (415/492) and 66.6% (209/314) of patients with monoinfection were gametocytemic at enrollment, respectively. The ratio of gametocytemia to asexual parasitemia did not differ between acute and recurrent infections (*P* = .48 in Thailand, *P* = .08 in Indonesia). High asexual parasitemia was associated with an increased risk of gametocytemia during follow-up in both locations. In Thailand, the cumulative incidence of gametocytemia between day 7 and day 42 following dihydroartemisinin + piperaquine (DHA + PIP) was 6.92% vs 29.1% following chloroquine (*P* < .001). In Indonesia, the incidence of gametocytemia was 33.6% following artesunate + amodiaquine (AS + AQ), 7.42% following artemether + lumefantrine, and 6.80% following DHA + PIP (*P* < .001 for DHA + PIP vs AS + AQ).

***Conclusions.*** *P. vivax* gametocyte carriage mirrors asexual-stage infection. Prevention of relapses, particularly in those with high asexual parasitemia, is likely the most important strategy for interrupting *P. vivax* transmission.

*Plasmodium vivax* threatens almost half of the world's population and is associated with significant, relapsing morbidity [1–3]. It is set to become the dominant malaria species in the Asia–Pacific region [2]. Transmission of *P. vivax* is dependent upon development of sufficient densities of mature, infectious gametocytes in the peripheral circulation and their subsequent ingestion by competent *Anopheles* mosquito vectors. Designing effective intervention strategies that will reduce the chance that transmission occurs requires a comprehensive understanding of the biological and epidemiological attributes of *P. vivax* gametocytes. We analyzed data from 3 large randomized controlled trials (1 on the Thai–Myanmar border [4] and 2 in Papua, Indonesia [5, 6]) to determine and compare the demographic and clinical factors associated with patent gametocytemia on presentation with vivax malaria and during the 6–9 weeks following treatment with 1 of 4 antimalarial regimens: artemether + lumefantrine (AM + LUM), dihydroartemisinin + piperaquine (DHA + PIP), artesunate + amodiaquine (AS + AQ), and chloroquine monotherapy (CQ).

## METHODS

### Study Sites

#### Thailand

The single Thai study in this analysis was conducted at Shoklo Malaria Research Unit clinics along the northwestern border of Thailand [4]. This region has low, seasonal malaria transmission with an incidence of approximately 1 episode per person-year, 53% due to *P. vivax* and 37% due to *Plasmodium falciparum* [7, 8]. *P. vivax* relapses occur approximately 3–4 weeks following administration of rapidly eliminated antimalarials [9].

#### Papua, Indonesia

The 2 Indonesian studies included in this analysis took place at the same 2 clinics in the municipality of Timika in south–central Indonesia, eastern Indonesia [5, 6]. The demographics and geography of this region have been described previously [3, 10]. In 2005, the prevalence of asexual parasitemia was 6.4% for *P. vivax* and 7.5% for *P. falciparum* [10]. Local *P. vivax* strains relapse at intervals of approximately 3 to 4 weeks [5, 6].

### Study Designs

The Thai study was carried out between January 2007 and December 2008 and compared DHA + PIP with CQ for slide-confirmed uncomplicated *P. vivax* monoinfections [4]. Primaquine was not given. Pregnant or lactating women; patients aged <1 year or <5 kg in weight; and those with known hypersensitivity to the study medications, intercurrent illness, or a hematocrit <20% were excluded.

The first of the 2 Indonesian studies was carried out between July 2004 and June 2005 and compared DHA + PIP with AM + LUM for slide-confirmed uncomplicated malaria due to *P. falciparum, P. vivax*, or mixed species infection [5]. Unsupervised primaquine at a dose of 0.3 mg base/kg per day for 14 days was prescribed for patients with *P. vivax* and mixed species infections at day 28 if they did not have glucose-6-phosphate dehydrogenase (G6PD) deficiency.

The second of the 2 Indonesian studies was carried out between July 2005 and December 2005 and compared DHA + PIP with AS + AQ for the treatment of slide-confirmed uncomplicated *P. falciparum, P. vivax*, or mixed species malaria [6]. Unsupervised primaquine was offered to G6PD-normal individuals with *P. vivax* or mixed species malaria immediately after completion of the study regimens. Patients who were pregnant or lactating were excluded from both studies as were those who had a parasitemia of >4% or who fulfilled World Health Organization criteria for severe malaria [11]. The study comparing DHA + PIP with AL + LUM excluded individuals who weighed <10 kg, whereas the study of DHA + PIP vs AS + AQ excluded individuals who weighed <5 kg or were aged <1 year. Details of the drug regimens can be found in the respective study publications [4–6].

In all studies, patients were followed with daily symptom enquiry and physical examination as well as blood smears until aparasitemic and afebrile. Thereafter, patients were followed weekly for 6 weeks (42 days) in Indonesia and 9 weeks (63 days) in Thailand. Block randomization and allocation concealment using sealed opaque envelopes were used in all studies. Drug administration was open label, but microscopists at both sites were unaware of patient allocation.

### Laboratory Methods

In Thailand, sexual and asexual parasite counts, including the individual densities of trophozoites and schizonts, were expressed per 500 white blood cells (WBCs); if parasitemia was >1%, densities were expressed per 1000 red blood cells. Slides were declared negative after examination of at least 100 high-power fields. Hematocrit was measured using a microcentrifuge (Hawksley) and, in this analysis, converted to a hemoglobin concentration in g/dL by multiplying the percentage by a factor of 0.34 [12].

In Indonesia, asexual and sexual parasite counts were measured on Giemsa-stained thick films and reported per 200 WBCs. Slides were declared negative after examination of at least 100 high-power microscope fields. A thin film was also examined to confirm parasite species and for quantification per 1000 red blood cells if the parasitemia was >200 per 200 WBCs. Hemoglobin was measured using a portable photometer (HemoCue Hb201+, Angelholm, Sweden). G6PD status was tested using the fluorescent spot test in both locations.

### Statistical Analysis

The primary outcomes of interest were the presence or absence of *P. vivax* gametocytemia at enrollment, time to clearance of *P. vivax* gametocytes, and appearance of *P. vivax* gametocytes up to 63 days following enrollment. All analyses were stratified by country because there were several sources of intercountry heterogeneity, including differences in slide-reading protocols and likely differences in preexisting immunity to *P. vivax* malaria. The following were defined a priori as potential risk factors for gametocytemia at enrollment: sex, age (<5 years, 5 to <15 years, ≥15 years), G6PD status (normal or abnormal), asexual *P. vivax* parasite density (log_e_ [parasites/µL]), anemia (hemoglobin <9 g/dL), fever (temperature >37.5°C), species of infection at enrollment (pure *P. vivax* vs mixed *P. vivax/P. falciparum* infection [Indonesia only]), and stage of infection at enrollment (presence or absence of schizonts [Thailand only]). Risk factors for the appearance of *P. vivax* gametocytes during follow-up were as above plus clearance of asexual parasitemia by day 1 (yes, no) and antimalarial regimen.

Analyses of gametocytemia at enrollment were done using separate univariable logistic regression models for the 2 locations. All patient factors were subsequently included in separate multivariable logistic regression models for each location. In Indonesia, there was no difference in gametocyte carriage following DHA + PIP in the first study (in which unsupervised primaquine was prescribed at day 28) compared with the second study (in which unsupervised primaquine was prescribed at day 3). Results from the 2 Indonesian studies were therefore pooled in all analyses.

The cumulative incidence of *P. vivax* gametocytemia in each location between day 7 and the end of follow-up was assessed for each antimalarial regimen using the Kaplan-Meier method and compared using the log-rank test. Clinical and demographic risk factors for recurrent gametocytemia were examined using univariable and multivariable Cox regression models for each location (the latter stratified by treatment group). Fulfillment of the proportional hazards assumption was assessed by comparing log (cumulative hazard) by time of follow-up curves and subsequently by examination of Schoenfeld residuals. Patients who had recurrent asexual *P. vivax* infection without concurrent gametocytemia were retreated with antimalarial medication and were therefore censored at the time of recurrence. The proportions of individuals who had cleared their gametocytes by day 1 and day 2 were examined for each regimen stratified by location and compared using the *χ*² test or Fisher exact test. Comparisons of nonnormal distributions were made using the Mann–Whitney *U* test or the Wilcoxon signed rank test for matched data. We explored the association between asexual and sexual *P. vivax* parasite density (log_e_ transformed) using Pearson correlation coefficient. All data merging and analyses were done using STATA version 10.1 (College Station, Texas).

### Ethical Clearance

The ethics committees of the Faculty of Tropical Medicine, Mahidol University, Thailand, and the Oxford Tropical Research, United Kingdom, approved the Thai study. The ethics committees of the National Institute of Health Research and Development, Indonesia, and the Menzies School of Health Research, Australia, approved the Indonesian studies.

## RESULTS

A total of 492 patients with *P. vivax* monoinfections were evaluable in the Thai dataset and 476 patients with *P. vivax* infections (314 with monoinfections and 162 with concurrent *P. falciparum* infections) were evaluable in the Indonesian dataset (Table 1). Patients enrolled in the Thai study were slightly older and less anemic than their Indonesian counterparts and had higher asexual parasitemias (median = 6565/µL vs 2595/µL, *P* < .001). Results relevant to the patients with mixed infection in Indonesia are presented in a separate subsection below.

**Table 1.** Characteristics of Evaluable Patients in the Thai and Indonesian Studies

Characteristic	Thailand	Indonesia (monoinfections)	Indonesia (mixed infections)
N	492	314	162
Sex			
Male	328 (66.7%)	169 (53.8%)	103 (63.6%)
Age			
<5 y	66 (13.4%)	86 (27.4%)	26 (16.0%)
5 to <15 y	135 (27.4%)	78 (24.8%)	44 (27.2%)
>15 y	291 (59.1%)	150 (47.8%)	92 (56.8%)
G6PD status			
Normal	463 (94.1%)	231 (73.6%)	104 (64.2%)
Abnormal	28 (5.7%)	45 (14.3%)	12 (7.4%)
Febrile (>37.5°C)	166 (33.7%)	52 (16.6%)	62 (38.3%)
Asexual *Plasmodium vivax* parasitemia (/µL)	6565 (193–30 551)^a^	2595 (140–27 500)^a^	606 (38–14 036)^a^
*P. vivax* gametocytes detected	415 (84.3%)	209 (66.6%)	92 (56.8%)
*P. vivax* gametocytemia (per µL)	266 (33–2158)^a^	113 (35–727)^a^	98 (21–1205)^a^
Hemoglobin (g/dL)	12.6 (9.9–15.6)^a^	10.3 (6.3–14.5)^a^	9.8 (5.6–14.4)^a^
Anemia (Hb <9 g/dL)	5 (1.0%)	94 (29.9%)	65 (40.1%)
Stage of infection			
Trophozoites alone	339 (68.9%)	( … )	( … )
Trophozoites and schizonts	153 (31.1%)	( … )	( … )
Treatment			
Artemether + lumefantrine	0 (0%)	84 (26.8%)	58 (35.8%)
Dihydroartemisinin + piperaquine	248 (50.4%)	169 (53.8%)	86 (53.1%)
Artesunate + amodiaquine	0 (0)	61 (19.4%)	18 (11.1%)
Chloroquine	244 (49.6%)	0 (0%)	0 (0%)

Abbreviation: G6PD, glucose-6-phosphate dehydrogenase.

^a^ Median (5th–95th percentiles).

### Gametocytemia on Enrollment

**Figure 1.**
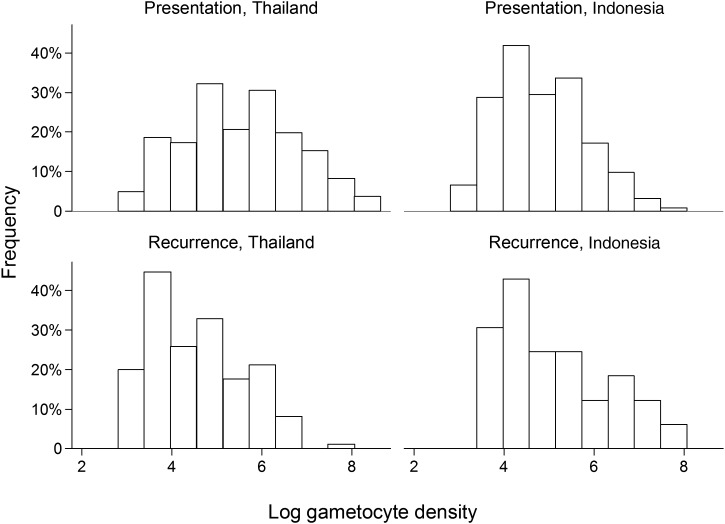
Frequency distribution of log_e_ gametocyte density for those with *Plasmodium vivax* monoinfections on presentation for treatment and at the time of recurrence.

Gametocytes were detected on enrollment in 84.3% (415/492) of patients in Thailand and 66.6% (209/314) of patients with *P. vivax* monoinfections in Indonesia (*P* < .001). Thai patients also had a higher median gametocyte density than Indonesian patients (*P* < .001; Table 1 and Figure 1).

In univariable analyses, higher log_e_ asexual parasite density was associated with a highly statistically significant increase in the risk of gametocytemia on presentation in both locations (Table 2). Presence of schizonts on the admission blood film was associated with a 14-fold increased risk of gametocytemia in Thailand (*P* < .001). In multivariable analyses, the only independent predictors of gametocytemia on presentation were higher asexual parasitemia (adjusted odds ratio [AOR] per log_e_ order increase = 2.31; 95% confidence interval [CI], 1.86–2.86; *P* < .001 in Thailand and AOR = 1.61; 95% CI, 1.39–1.87; *P* < .001 in Indonesia) and schizontemia at enrollment (AOR = 6.31; 95% CI, 1.78–22.4; *P* = .004 [Thailand only]).

**Table 2.** Risk Factors for *Plasmodium vivax* Gametocytemia at Presentation in Patients Enrolled in the Thai and Indonesian Trials

	Univariable Models						Multivariable Models			
	Thailand		Indonesia (monoinfection)		Indonesia (mixed infection)		Thailand		Indonesia	
Risk Factor	OR (95% CI)	*P* Value	OR (95% CI)	*P* Value	OR (95% CI)	*P* Value	AOR (95% CI)	*P* Value	AOR (95% CI)	*P* Value
Sex										
Male	1.00		1.00		1.00		1.00		1.00	
Female	0.85 (.51–1.42)	.54	1.03 (.64–1.66)	.90	1.20 (.62–2.31)	.58	0.97 (.50–1.88)	.93	0.91 (.58–1.44)	.70
Age										
<5 y	1.16 (.51–2.60)	.73	0.86 (.48–1.54)	.62	0.76 (.32–1.84)	.55	1.22 (.44–3.45)	.70	0.82 (.45–1.49)	.51
5 to <15 y	0.58 (.34–.99)	.05	0.54 (.30–.95)	.03	0.75 (.36–1.56)	.45	0.72 (.36–1.46)	.36	0.59 (.34–1.02)	.06
≥15 y	1.00		1.00		1.00		1.00		1.00	
G6PD status										
Normal	1.00		1.00		1.00		1.00		1.00	
Abnormal	0.85 (.31–2.30)	.75	1.51 (.74–3.07)	.26	1.11 (.33–3.73)	.87	1.11 (.30–4.03)	.88	1.31 (.68–2.53)	.42
Log_e_ asexual parasite density (per log_e_ order increase)	2.36 (1.95–2.85)	<.001	1.49 (1.27–1.74)	<.001	2.01 (1.57–2.58)	<.001	2.31 (1.86–2.86)	<.001	1.61 (1.39–1.87)	<.001
Anemia (Hb <9 g/dL)	0.75 (.08–6.76)	.79	1.89 (1.08–3.28)	.03	1.35 (.71–2.57)	.36	1.26 (.08–19.6)	.87	1.39 (.83–2.34)	.21
Fever (>37.5°C)	1.10 (.64–1.87)	.74	1.59 (.81–3.13)	.18	1.11 (.58–2.11)	.76	0.53 (.27–1.05)	.07	0.89 (.50–1.58)	.69
Stage of infection										
Trophozoites alone	1.00		( … )		( … )		1.00		( … )	
Trophozoites and schizonts	14.0 (4.33–45.1)	<.001	( … )		( … )		6.31 (1.78–22.4)	.004	( … )	
Species of infection										
*P. vivax* monoinfection	( … )		( … )		( … )		( … )		1.00	
Mixed *P. vivax / P. falciparum*	( … )		( … )		( … )		( … )		1.03 (.86–1.24)	.74

Abbreviations: AOR, adjusted odds ratio; CI, confidence interval; G6PD, glucose-6-phosphate dehydrogenase; OR, odds ratio.

### Gametocyte Clearance

**Figure 2.**
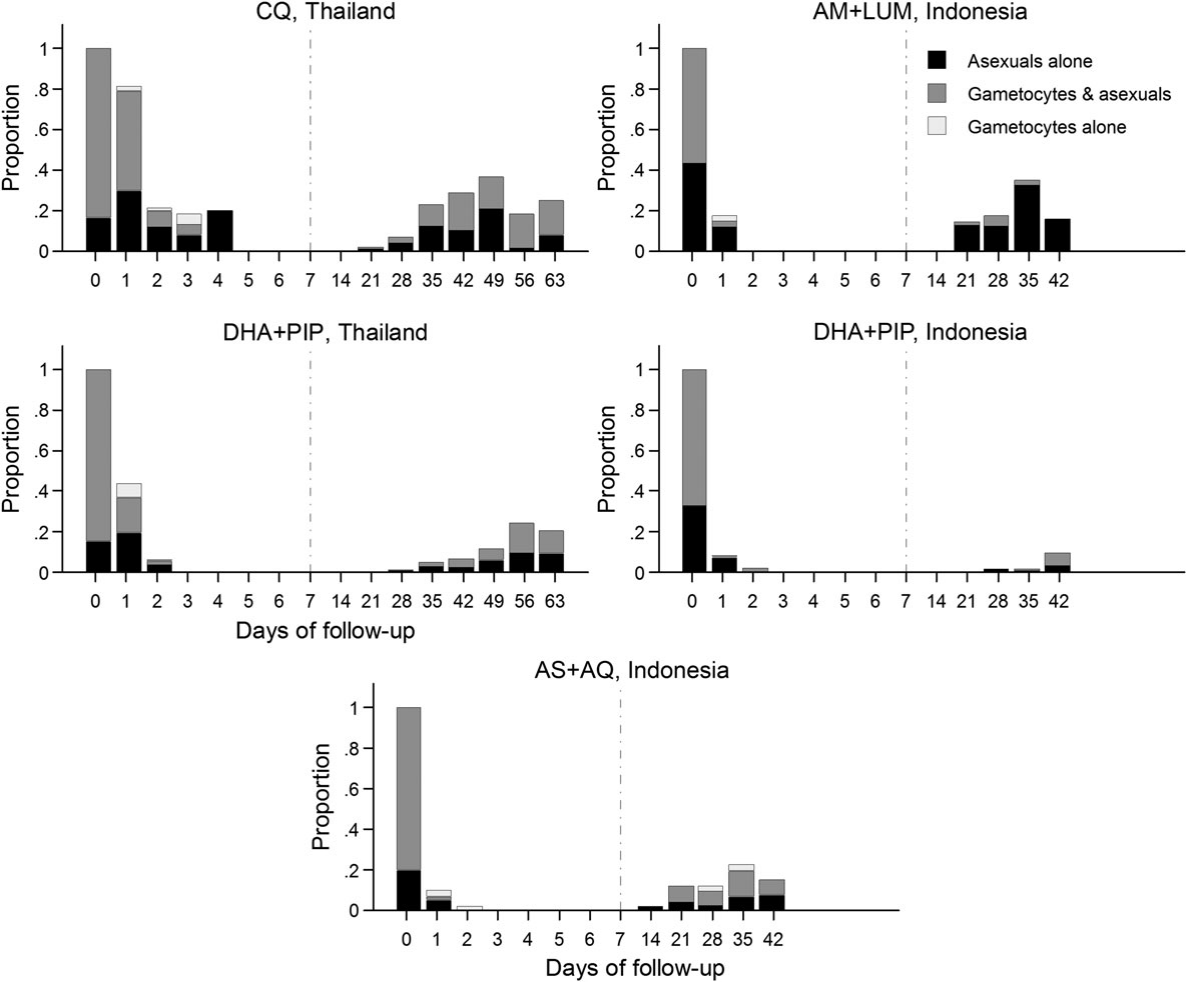
Proportion of individuals examined with sexual and/or asexual forms of *Plasmodium vivax* from presentation through to end of follow-up in Thailand and Indonesia (excludes patients with mixed infection on presentation in Indonesia). Abbreviations: AM + LUM, artemether + lumefantrine; AS + AQ, artesunate + amodiaquine; CQ, chloroquine; DHA + PIP, monotherapy dihydroartemisinin + piperaquine.

Overall, 42.5% (207/487) of patients in Thailand had cleared their asexual parasitemia by day 1 vs 90.7% (262/289) of patients with *P. vivax* monoinfections in Indonesia (*P* < .001). Of those with gametocytemia on enrollment, 58.4% (240/411) had cleared their gametocytemia by day 1 in Thailand vs 96.4% (270/280) in Indonesia (*P* < .001). If gametocytemia had been established against 200 rather than 500 white cells in Thailand, an estimated 78 additional patients with gametocytemia on day 1 would have been classified as agametocytemic and the proportion of patients who had cleared gametocytemia by day 1 would have been 77.4% as opposed to 58.4%. The proportions of patients who had cleared their gametocytemia by day 1 for the individual drugs in Thailand were 73.4% (152/207) after DHA + PIP vs 43.1% (88/204) after CQ, *P* < .001. By day 2, 93.5% (245/262) of patients had cleared their gametocytes in Thailand and by this time there was no difference between treatment arms. In Indonesia, the proportions of patients who had cleared their gametocytemia by day 1 were 90.2% (37/41) following AM + LUM, 98.1% (101/103) following DHA + PIP, and 93.9% (46/49) following AS + AQ (*P* = .03 for AM + LUM vs DHA + PIP; *P* = .52 for AM + LUM vs AS + AQ; and *P* = .18 for DHA + PIP vs AS + AQ; Figure 2). No individuals at either site had persistent *P. vivax* gametocytes at day 7. In Thailand, 22.1% (17/77) of individuals without gametocytemia on admission developed *P. vivax* gametocytemia between day 1 and day 4; the risk being nonsignificantly greater following treatment with chloroquine (30.8% 12/39) than with DHA + PIP (13.2% 5/38); *P* = .06. In Indonesia, no patients (0/103) without gametocytemia on enrollment subsequently developed gametocytemia between day 1 and day 4 (*P* < .001).

### Gametocytemia During Follow-up

Overall, 146 of 492 (29.7%) participants had appearance of *P. vivax* gametocytemia between day 7 and day 63 in Thailand (67 [13.6%] of whom failed by day 42) and 28 of 314 (8.92%) participants with *P. vivax* monoinfections had appearance of *P. vivax* gametocytemia between day 7 and day 42 in Indonesia (see Table 3). Of the 174 appearances of gametocytemia during follow-up, only 2 (1.15%) were not associated with concurrent asexual-stage infection; both individuals had been treated with AS + AQ. In Thailand, 54.2% (147/271) of patients had patent gametocytemia at the time of *P. vivax* asexual recurrence compared with 33.8% (26/77) following *P. vivax* monoinfection in Indonesia (*P* = .002).

**Table 3.** Cumulative Percentage Gametocyte Carriage by Treatment

	Cumulative Percentage Gametocyte Carriage (95% confidence interval)					*P* Value			
AM + LUM	DHA + PIP	AS + AQ	CQ	All	AM + LUM v DHA + PIP	AM + LUM v AS + AQ	DHA + PIP v AS + AQ	DHA + PIP v CQ
Day 7–42									
Thailand	( … )	6.92 (4.23–11.2)	( … )	29.1 (23.0–36.5)	16.9 (13.5–21.0)	( … )	( … )	( … )	<.001
Indonesia (pure)	7.42 (3.14–17.0)	6.80 (3.46–13.2)	33.6 (21.6–49.8)	( … )	12.1 (8.50–17.2)	.39	<.001	<.001	( … )
Indonesia (mixed)	17.5 (9.12–32.1)	4.76 (1.54–14.2)	34.7 (14.4–68.7)	( … )	12.3 (7.55–19.7)	.01	.39	.001	( … )
Day 7–63									
Thailand	( … )	32.9 (26.5–40.4)	( … )	57.9 (49.4–66.6)	43.7 (38.4–49.4)	( … )	( … )	( … )	<.001

Abbreviations: AM + LUM, artemether + lumefantrine; AS + AQ, artesunate + amodiaquine; CQ, chloroquine; DHA + PIP, dihydroartemisinin + piperaquine.

In Thailand, the day 42 cumulative risk of gametocyte carriage was lower following DHA + PIP (6.92%; 95% CI, 4.23%–11.2%) than following CQ (29.1%; 95% CI, 23.0%–36.5%; *P* < .001). The cumulative risk of gametocyte carriage by day 42 following *P. vivax* monoinfections in Indonesia was greatest for AS + AQ (33.6%; 95% CI, 21.6%–49.8%) and lowest for DHA + PIP (6.80%; 95% CI, 3.46%–13.2%; *P* < .001; Table 3). There was no difference in the day 42 cumulative risk of gametocytemia following DHA + PIP between the Thai and Indonesian studies (Table 3).

In univariable models, risk factors for appearance of gametocytes during follow-up included higher initial asexual parasite density in both locations and presence of gametocytemia on enrollment, as well as persistence of asexual parasitemia on day 1 in Thailand (Table 3). Persistent asexual parasitemia on day 2 was rare in Indonesia; in Thailand, it was not associated with recurrent gametocytemia in a univariable model (hazard ratio = 1.47; 95% CI, .87–2.49; *P* = .15). After adjusting for confounding factors, higher asexual parasite density on enrollment was associated with a greater chance of recurrent gametocytemia in both Thailand and Indonesia (adjusted hazard ratio [AHR] = 1.18; 95% CI, 1.02–1.35; *P* = .02 in Thailand and AHR = 1.58; 95% CI, 1.25–1.98; *P* < .001 in Indonesia; Table 4).

**Table 4.** Risk Factors for Gametocytemia During Follow-Up

Risk Factor			Univariable Models				Multivariable Models			
	Thailand, day 7–63		Indonesia (monoinfection), day 7–42		Indonesia (mixed infection), day 7–42		Thailand, day 7–63		Indonesia, day 7–42	
	HR (95% CI)	*P* Value	HR (95% CI)	*P* Value	HR (95% CI)	*P* Value	AHR (95% CI)	*P* Value	AHR (95% CI)	*P* Value
Female sex	0.93 (.66–1.31)	.69	2.21 (1.02–4.78)	.05	1.16 (.41–3.27)	.77	0.91 (.62–1.32)	.62	1.62 (.83–3.18)	.16
Age										
<5 y	1.53 (.98–2.39)	.06	2.28 (.96–5.40)	.06	0.93 (.20–4.36)	.92	1.54 (.95–2.49)	.08	1.41 (.58–3.42)	.45
5 to <15 y	0.78 (.53–1.16)	.23	1.39 (.52–3.74)	.51	1.29 (.42–3.95)	.65	0.89 (.58–1.35)	.57	1.92 (.82–4.52)	.13
≥15 y	1.00		1.00		1.00		1.00		1.00	
G6PD status										
Normal	1.00		1.00		^a^		1.00		1.00	
Abnormal	1.05 (.49–2.25)	.90	1.56 (.66–3.67)	.31	^a^		0.95 (.43–2.09)	.90	1.84 (.74–4.53)	.19
Enrollment log_e_ asexual parasite density (per log_e_ order increase)	1.21 (1.08–1.36)	.001	1.48 (1.14–1.91)	.003	1.72 (1.30–2.27)	<.001	1.18 (1.02–1.35)	.02	1.58 (1.25–1.98)	<.001
Gametocytes on enrollment	1.82 (1.07–3.11)	.03	2.15 (.87–5.31)	.10	2.22 (.71–6.98)	.17	1.22 (.68–2.22)	.50	1.31 (.54–3.16)	.55
Persistent asexual parasitemia on day 1	1.45 (1.04–2.03)	.03	2.12 (.73–6.17)	.17	1.78 (.39–8.01)	.46	0.84 (.57–1.25)	.39	1.13 (.42–3.01)	.81
Anemia on enrollment (Hb <9 g/dL)	0.54 (.08–3.86)	.54	1.46 (.68–3.17)	.33	1.68 (.59–4.81)	.33	0.85 (.11–6.45)	.88	1.26 (.60–2.62)	.55
Fever on enrollment (>37.5°C)	1.33 (.94–1.87)	.11	0.40 (.09–1.67)	.21	1.12 (.40–3.15)	.83	1.22 (.84–1.77)	.29	0.45 (.17–1.20)	.11
Schizonts on admission blood film	1.23 (.87–1.73)	.24	( … )		( … )		1.05 (.70–1.57)	.81	( … )	
Species at enrollment										
*Plasmodium vivax* monoinfection	( … )		( … )		( … )		( … )		1.00	
Mixed *P. vivax / Plasmodium falciparum*	( … )		( … )		( … )		( … )		2.76 (1.26–6.04)	.01

Abbreviations: AHR, adjusted hazard ratio; CI, confidence interval; G6PD, glucose-6-phosphate dehydrogenase; HR, hazard ratio.

Multivariable models stratified by treatment group.

^a^ No patients with mixed infection and an abnormal G6PD status had a recurrence of *P. vivax* gametocytemia between 7 and 42 days.

**Figure 3.**
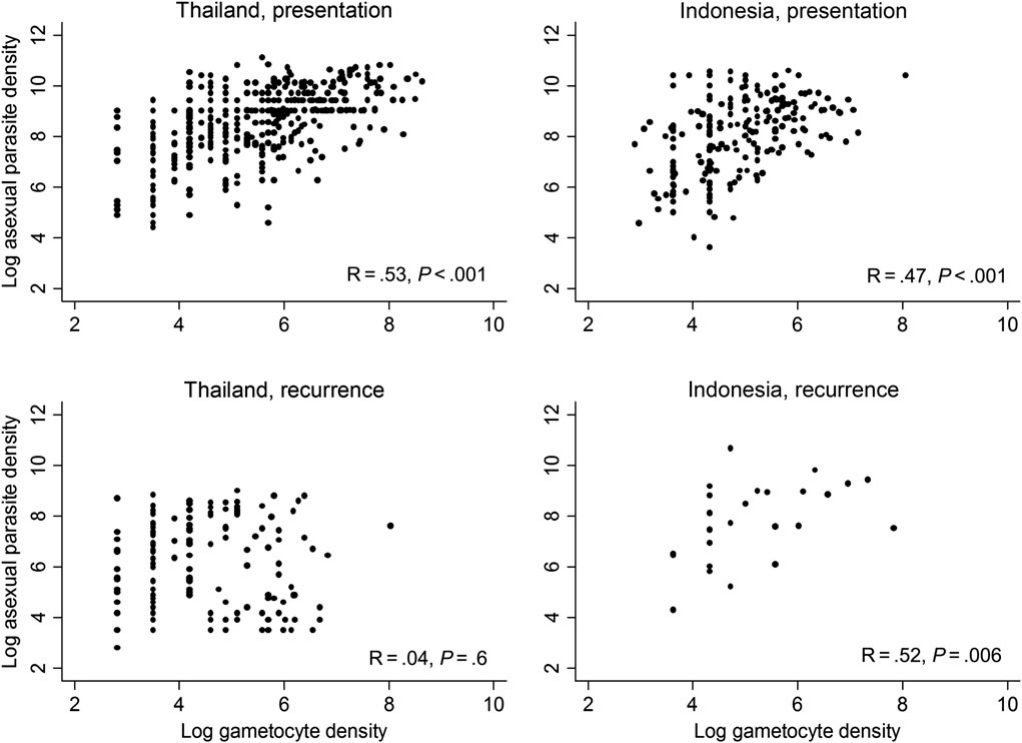
Correlation between the log_e_ density of asexual and sexual stages of *Plasmodium vivax* at presentation for treatment and at the time of recurrence after treatment (analyses limited to those with *P. vivax* monoinfections at enrollment).

The relationship between log_e_ transformed asexual- and sexual-stage density at enrollment and during follow-up is presented in Figure 3. In Thailand, the median ratio of gametocytes to asexuals did not differ between enrollment and recurrence (median ratio at enrollment = 0.04, interquartile range [IQR] = 0.009–0.08; median ratio at recurrence = 0.01, IQR = 0–0.19, *P* = .49). The same was true in Indonesia (median ratio at enrollment = 0.015, IQR = 0–0.076; median ratio at recurrence = 0, IQR = 0–0.03, *P* = .08).

### Mixed *P. vivax*/*P. falciparum* Infections

In Indonesia, patients with mixed infections on enrollment were less likely to have patent *P. vivax* gametocytemia than patients with *P. vivax* monoinfection (56.8% [92/162] vs 66.6% [209/314], *P* = .04). However, those with mixed infection were at significantly greater risk of recurrent gametocytemia between day 7 and day 42 compared with patients with *P. vivax* monoinfection (AHR = 2.76; 95% CI, 1.26–6.04; *P* = .01).

## DISCUSSION

Our analysis of 3 large clinical drug trials from Thailand and Indonesia highlights several fundamental properties of *P. vivax* transmission dynamics, some of which have been given little consideration since early studies of neurosyphilitics and military personnel in the first half of the 20th century [13–21]. First, patent *P. vivax* gametocytemia is present in the majority of patients by the time they seek treatment. Second, *P. vivax* gametocytes do not persist after asexual parasite clearance (no patient in either country had persistent gametocytemia at day 7). Third, the relationship between asexual- and sexual-stage parasitemia does not differ substantially between initial and recurrent infections. Fourth, there are significant differences in the effects of artemisinin combination regimens on the risk of recurrent parasitemia and therefore the short-term transmissibility of *P. vivax* infections.

In symptomatic falciparum malaria, patent gametocytemia occurs after the onset of symptoms and usually during convalescence [22, 23]. Rapidly effective blood schizontocidal drugs can therefore have a profound impact on overall gametocyte carriage and transmission potential [24]. The artemisinin derivatives are highly potent antimalarials that reduce the biomass of asexual parasites rapidly while also exerting strong gametocytocidal activity against early-stage sexual forms [24–28]. When combined with a slowly eliminated partner drug, the artemisinin derivatives minimize the risk of recrudescence and reduce *P. falciparum* transmissibility [29].

The dynamics of gametocyte carriage in vivax malaria are notably different [30]. Sexual stages appear early in the course of infection [13–16, 19, 23, 31] together with the rise in asexual parasitemia; thus, transmission often occurs before antimalarial treatment. Unlike *P. falciparum* gametocytes, *P. vivax* sexual forms are susceptible to all blood schizontocidal medications [32]. The relative transmission-blocking benefit of drugs with greater potency, such as the artemisinin derivatives that reduce *P. vivax* parasitemia more rapidly than others, is likely to be minimal. There are 2 reasons for this. First, gametocytes are often present for several days before presentation and are therefore likely to be transmitted prior to treatment [33, 34]. Assuming complete parasitological cure, administration of highly potent artemisinin-based therapy instead of chloroquine will truncate gametocyte carriage associated with the initial episode by at best 24–48 hours. Second, *P. vivax* infection is associated with multiple relapses, each associated with gametocytemia and thus transmissible for several days prior to clinical detection. Preventing recurrence, in particular due to relapse, is thus more important for reducing transmission of vivax malaria than rapid removal of gametocytes at each clinical presentation.

The antimalarial regimen with the greatest potential to block transmission of *P. vivax* will include a highly active blood schizontocidal regimen that completely eradicates blood stages and thus prevents recrudescence in combination with a hypnozoitocidal medication for preventing future relapses. Unfortunately, toxicity concerns and poor adherence to 2-week regimens continue to hamper the safe and effective use of primaquine, the only currently licensed hypnozoitocidal drug [35]. Where primaquine is not used or has been shown to be ineffective, slowly eliminated blood schizontocides that suppress the first relapse may have benefits over regimens with shorter elimination half-lives [36], though whether they reduce the total number of relapses and overall transmission potential is unknown.

AS + AQ has consistently been associated with higher *P. falciparum* recrudescence and, as shown in this analysis, higher *P. vivax* recurrence rates than either AM + LUM or DHA + PIP [6, 37]. This is likely to be attributable to the relatively short elimination half-life of amodiaquine and declining parasite susceptibility to this drug [6]. Gametocyte carriage was higher following AM + LUM (half-life approximately 4 days) than DHP + PIP, although this only reached significance in patients treated for mixed infections (Table 3).

Chloroquine is potent against susceptible *P. vivax* strains and has an elimination half-life of 1–2 months [38]. It therefore has the potential to limit recrudescence and suppress the first and possibly even second *P. vivax* relapse. In Thailand, chloroquine was associated with greater gametocyte carriage during follow-up compared with DHA + PIP (elimination half-life approximately 28 days), suggesting declining chloroquine susceptibility of local strains. This scenario is likely to be mirrored in other regions where chloroquine has been used as the mainstay of vivax malaria treatment for many years [36].

High asexual parasite density was shown to be a strong risk factor for gametocyte carriage during follow-up, independent of age and other potential confounders. There are 2 likely explanations for this finding. First, high asexual parasitemia is associated with an increased risk of parasite recrudescence (as shown in falciparum malaria) [29, 39–41]. Second, high parasite density reflects poor immunity, which has been associated with a greater risk of patent relapse [42]. Since *P. vivax* gametocytemia mirrors asexual infection, a higher risk of recurrent asexual infection, whether due to recrudescence or relapse, will result in a higher risk of gametocyte carriage. In other words, the factors that determine *P. vivax* transmissibility are those that determine asexual-stage parasite dynamics.

Patients with mixed *P. vivax/P. falciparum* infections in Indonesia were at greater risk of recurrent gametocytemia than those with vivax monoinfections. This contrasts with mixed species asexual infections in Thailand, which have been associated with a lower risk of *P. falciparum* gametocytemia [29]. In Indonesia, mixed infections are more severe than monoinfections with either species [3]. Malarial illness has been hypothesized to precipitate *P. vivax* relapses [9, 43, 44]. Therefore, the increased risk of recurrent gametocytemia following mixed infection may relate to greater pathophysiological derangement and hence greater stimulation of dormant liver-stage parasites. Alternatively, mixed infection in Indonesia may reflect poor immunity, which in turn is associated with a greater risk of relapse. As we made multiple comparisons, the possibility of a chance finding must also be considered.

Our analysis has limitations. Follow-up was 3 weeks longer in Thailand than in Indonesia and thus conclusions drawn for the 42- to 63-day periods were based on Thai data only. A 42-day follow-up is insufficient to capture first relapses that follow administration of slowly eliminated antimalarial drugs. Parasite counts were done against 200 WBCs in Indonesia whereas in Thailand they were done against 500 WBCs. This will have increased the likelihood of gametocyte detection in Thailand relative to Indonesia and may partially explain the shorter gametocyte clearance times in Indonesia. Preexisting immunity to *P. vivax* is likely to have been greater in Indonesia than in Thailand due to more intense parasite exposure. This may have contributed to the slower gametocyte clearance times in Thailand.

Unsupervised primaquine was prescribed for individuals with normal G6PD activity at day 28 in the first Indonesian study and at day 3 in the second Indonesian study. Exploratory analyses revealed that this difference did not have any substantial effect on subsequent gametocyte carriage. Residual minor effects will have been controlled for by inclusion of G6PD status in the multivariable models.

Both *P. vivax* and *P. falciparum* can be transmitted at subpatent gametocyte densities [19, 45–50]. Thus, microscopic quantification may have underestimated the total period of infectiousness following therapy. Without treatment, *P. vivax* gametocytes are reported to persist in the peripheral circulation for a maximum of 3 days [48]. Since the study drugs reduced parasitemia rapidly, any persisting period of infectiousness from subpatent gametocytemia will be short-lived in the absence of recrudescence.

In conclusion, we have shown that *P. vivax* gametocytemia closely mirrors asexual-stage carriage. Persistence of patent gametocytemia following eradication of asexual stages does not occur. Our results indicate that the most important means of blocking *P. vivax* transmission is likely to be prevention of future relapses, especially in patients with high asexual parasite density and mixed infections. Optimal prescribing practices that maximize patient adherence to primaquine are needed and, given the limitations of this drug, very high priority must be given to the development of novel antirelapse strategies.
